# Lipid Isolated from a *Leishmania donovani* Strain Reduces *Escherichia coli* Induced Sepsis in Mice through Inhibition of Inflammatory Responses

**DOI:** 10.1155/2014/409694

**Published:** 2014-07-09

**Authors:** Subhadip Das, Nabanita Chatterjee, Dipayan Bose, Somenath Banerjee, Prajnamoy Pal, Tarun Jha, Krishna Das Saha

**Affiliations:** ^1^Cancer Biology & Inflammatory Disorder Division, CSIR-Indian Institute of Chemical Biology, 4 Raja S.C. Mullick Road, Kolkata, West Bengal 700032, India; ^2^Department of Chemistry, Fakir Chand College, South 24 Parganas, Diamond Harbour, West Bengal 743331, India; ^3^Division of Medicinal and Pharmaceutical Chemistry, Department of Pharmaceutical Technology, Jadavpur University, P.O. Box 17020, Kolkata 700032, India

## Abstract

Sepsis is the reflection of systemic immune response that manifests in the sequential inflammatory process in presence of infection. This may occur as a result of gram-negative bacterial sepsis including *Escherichia coli* infection that gives rise to excessive production of inflammatory mediators and causes severe tissue injuries. We have reported earlier that the lipid of attenuated *Leishmania donovani* suppresses the inflammatory responses in arthritis patients. Using heat killed *E. coli* stimulated macrophages, we have now investigated the effect of leishmanial total lipid (LTL) isolated from *Leishmania donovani* (MHO/IN/1978/UR6) for amelioration of the inflammatory mediators and transcriptional factor with suppression of TLR4-CD14 expression. To evaluate the *in vivo* effect, *E. coli* induced murine sepsis model was used focusing on the changes in different parameter(s) of lung injury caused by sepsis, namely, edema, vascular permeability, and pathophysiology, and the status of different cytokine-chemokine(s) and adhesion molecule(s). Due to the effect of LTL, *E. coli* induced inflammatory cytokine-chemokine(s) levels were significantly reduced in serum and bronchoalveolar lavage fluid simultaneously. LTL also improved the lung injury and suppressed the cell adhesion molecules in lung tissue. These findings indicate that LTL may prove to be a potential anti-inflammatory agent and provide protection against gram-negative bacterial sepsis with pulmonary impairment.

## 1. Introduction

The consequences of a complex immune reaction are described as sepsis that represents an uncontrolled inflammatory outburst from a harmful host response to infection [1] causing disruption and damage to several cells and tissues [2]. Macrophages, key players of the immune system, play an important role in the pathogenesis of inflammation. They secrete various inflammatory mediators such as prostaglandins, reactive oxygen, and nitrogen species, inflammatory cytokines including tumor necrosis factor alpha (TNF-*α*), interleukin-1 (IL-1), interleukin-6 (IL-6), interleukin-10 (IL-10), interleukin-12 (IL-12), and interleukin-17 (IL-17), chemokines including macrophage inflammatory protein (MIP), and bioactive lipids. These are regulated by the ubiquitous transcription factor, nuclear factor *κ*B (NF-*κ*B) [3, 4]. I*κ*B appears to function as a strong negative feedback mechanism that allows a fast turn-off of the NF-*κ*B response to control inflammation associated diseases [5, 6].

Though different bacteria have been identified as causative organisms in sepsis, gram-negative bacteria like* Escherichia coli* remain as one of the most common pathogens (up to 60%) in intraperitoneal infections with high mortality rates [7, 8]. Moreover, the recognition of CD14-TLR4 complex by cell wall components of gram-negative bacteria (*E. coli*) may lead to activation of the inflammatory responses [9]. The overproduction of inflammatory cytokines generates systemic activation which affects vascular permeability and gives rise to metabolic changes that can lead to tissue injury and eventually to the failure of various major organs to induce mortality [10]. Infectious inflammatory stimuli elicit acute lung distress [11] which may be perceived as the most fatal cause effecting the initiation of various cellular cascades. It may also lead, firstly, to preeminence of inflammatory cells in the interstitium and alveolar spaces and, secondly, to an increase in PMN-derived proteases and oxidative metabolites in the bronchoalveolar lavage fluid (BALF) [12]. Local inflamed cells in the lung interstitium activate the pulmonary capillary endothelium culminating in the expression of adhesion molecules on the endothelial cell [13]. It follows that strategies aimed at preventing cell activation may attenuate systemic inflammation relevant to lung injury [14, 15].

Microorganisms and their cellular component(s) may possess some degree of bioactivity, either against other microorganism(s) or against certain physiological states of a diseased body. It has been reported that sterile filtrates from* Clostridium histolyticum *and spores of* Clostridium tetani *induce tumor regression and can be used to treat cancers [16, 17]. Azurin, a protein produced by the pathogenic bacteria* P. aeruginosa*, induces apoptosis in cancer cells [18]. Myriocin isolated from the fungus* Isaria sinclairii* is an immunomodulating agent [19, 20]. It was reported that leishmanial lipids possess biological activity against stimulated macrophages and mammalian lymphocytes [21]. Recently we have shown that lipid from an attenuated strain of* Leishmania donovani* promastigote (MHO/IN/1978/UR6) suppresses several inflammatory mediators by inducing apoptosis in adherent synovial fluid mononuclear cells (SFMCs) of rheumatoid arthritis patients [22]. These findings encouraged us to evaluate the anti-inflammatory role of the leishmanial lipid against gram-negative bacteria (*E. coli*) induced inflammatory progression towards acute pulmonary damage. The present study reveals that leishmanial total lipid (LTL) has potent anti-inflammatory effect on gram-negative bacteria mediated inflammation both* in vitro* and* in vivo*.

## 2. Materials and Methods

### 2.1. Chemicals and Reagents

Roswell Park Memorial Institute medium (RPMI 1640), fetal bovine serum (FBS), and antibiotics were purchased from GIBCO BRL (Grand Island, NY). Culture plastic wares were obtained from NUNC (Roskilde, Denmark). Silica gel 60 HPTLC plates used were from E. Merck (Darmstadt, Germany). The TNF-*α* assay kit was procured from Amersham (NJ, USA) and PGE-2 kit from R & D system (MN, USA). IL-1*β*, IL-6, IL-10, IL-12p40, IL-17, and BD OptEIA assay kits were from BD Biosciences (USA), nitric oxide assay kit was from Calbiochem (Darmstadt, Germany), and goat anti-TNF-*α*, -IL-1*β*, -IL-6, -IL-10, -NF-*κ*B p65, -I*κ*B, -histone-H2B, -*β*-actin, -COX-2, -iNOS, -ICAM-1, -VCAM-1, -E-Selectin, -P-Selectin, and rabbit anti-CD14, -TLR4 were purchased from Santa Cruz Biotechnology (Santa Cruz, CA).

### 2.2. Isolation of Lipid from* Leishmania donovani *Promastigote Cells

Leishmania strain UR6 (MHO/IN/1978/UR6) was grown in Ray's modified medium [23] and the total lipid was isolated following the Bligh and Dyer method [24].

### 2.3. Thin-Layer Chromatography (TLC)

The leishmanial lipid was dissolved in 2/1 (v/v) chloroform-methanol. TLC was performed in chloroform-methanol-water (90/10/1) and lipid spots were visualized using iodine spray [22].

### 2.4. Murine Macrophage Cultures

Mouse peritoneal macrophages were obtained by lavage with 10 mL of cold Hank's balanced salt solution three days after intraperitoneal (i.p.) injection of 2 mL of 3% thioglycollate in saline (1.5 mL per mouse, Difco, Detroit, MI) [25]. Cells were maintained in RPMI-1640 supplemented with 10% (v/v) fetal calf serum and antibiotics (100 U/mL of penicillin, 100 *µ*g/mL of streptomycin), seeded at a density of 2 × 10^6^ cells/mL, and incubated at 37°C in a humidified 5% CO_2_ incubator to allow macrophage adherence.

### 2.5. Determination of Inflammatory Mediators by ELISA

The effect of LTL on inflammatory mediators including TNF-*α*, PGE2, IL-1*β*, IL-6, IL-17, IL-12p40, IL-10, and MIP-2 levels was investigated in heat-killed* E. coli* (O18:K1; 1 × 10^8^ CFU/mL) stimulated peritoneal macrophages and murine system as per the manufacturer's protocol.

### 2.6. Measurement of Cell Viability

Cell viability was evaluated using the MTT assay and absorption at 595 nm was measured by using an ELISA reader [26].

### 2.7. Extraction of Nuclear Proteins and Assay of NF-*κ*B p65

Cells were treated with either LTL [at a concentration of 50 *μ*g/mL (LTLd_1_) or 100 *μ*g/mL (LTLd_2_)] or left untreated, centrifuged, resuspended in 400 *µ*L of ice cold hypotonic buffer for 10 min, vortexed, and recentrifuged at 15,000 g at 4°C. Aliquots of the supernatant containing nuclear protein were added to incubation wells precoated with the NF-*κ*B p65 DNA-binding consensus sequence, and the translocated p65 subunit present in nuclear lysate was assayed [22].

### 2.8. Immunofluorescence Microscopy

The effect of LTL at the concentration of 100 *μ*g/mL (LTLd_2_) on* E. coli *stimulated NF-*κ*B activation with TLR4 and CD14 expression in mouse peritoneal macrophage cells was measured by immunocytochemical analysis. The cells, cultured on chambered plastic slides, were fixed with ethanol for 30 min at 4°C and the detergent was extracted with 0.3% Triton X-100 for 10 min at room temperature. After blocking with 3% bovine serum albumin (BSA) for 30 min, samples were incubated overnight with a primary antibody at 4°C. Samples were also incubated with FITC and TRITC conjugated secondary antibody for 2 h at room temperature. Nuclei were stained with DAPI and evaluated under an Andor spinning disc confocal microscope.

### 2.9. Western Blot Analysis

Cells and tissue protein lysates were analysed by standard western blotting procedure, using antibodies such as PGE2, iNOS, TNF-*α*, IL-1*β*, IL-6, IL-10, NF-*κ*B p65, ICAM-1, VCAM-1, P-selectin, and E-selectin obtained from Santa Cruz Biotechnology (Santa Cruz, CA) [27].

### 2.10. Animals

Sixteen-week-old female BALB/c mice (22–25 g) were obtained from the Indian Institute of Chemical Biology (Animal House). Experiments were done in adherence to the guidelines of the Institutional Animal Care and Use Committee.

### 2.11. Acute Toxicity Study of LTL

LTL were aseptically suspended in normal saline (0.9% NaCl solution) with 0.5% Tween 80 and administered intraperitoneally (i.p.) at a single dose of 1–500 mg/kg body wt in mice; each group consisted of six animals. The LD_50_ values were determined according to the method of Litchfield and Wilcoxon [28].

### 2.12. Induction of Sepsis and Survival Assay

*E. coli* O18:K1 was cultured in Luria-Bertani medium (Difco) at 37°C, harvested at midlog phase, and washed twice with sterile saline before injection to clear the bacteria of the medium. In all experiments mice were injected i.p. with heat-killed* E. coli* O18:K1, 10^4^ CFU in 200 *μ*L of sterile isotonic saline. For this study mice were divided into five groups (*n* = 10). The first group (control group) received vehicle only, the second group received only LTL, the third group received* E. coli*, and the remaining two groups received LTL at doses of 25 mg/kg (LTLD1) and 50 mg/kg (LTLD2) i.p. 2 h prior to administration of* E. coli*. Mice were monitored for survival twice a day for six days.

Blood samples (up to 300 *μ*L) were collected at time points 0, 1, 4, and 12 h after bacterial challenge by puncturing the orbital plexus, and serum samples were analyzed by ELISA according to the manufacturer's instructions [29, 30].

### 2.13. Histopathological Analysis and Scoring

After 24 h of* E. coli* challenge, the mice were sacrificed; lungs were collected from each group and stored in the fixative consisting of 10% paraformaldehyde at 4°C for 48 h. Hematoxylin-Eosin (H&E) and periodic acid-Schiff's (PAS) stainings were carried out according to the regular staining methods, and the slides were histopathologically evaluated using a semiquantitative scoring method. Lung injury was graded from 0 (normal) to 4 (severe) in four categories: interstitial inflammation, inflammatory cell infiltration, congestion, and edema. The total lung injury score was calculated by adding up the individual scores of each category [31, 32].

### 2.14. Immunohistochemistry

Paraffin-embedded blocks were cut into 5 *μ*m sections and mounted onto slides. The sections were deparaffinized with xylene and dehydrated over several series of alcohol. Antigen retrieval was performed by trypsin (0.05% trypsin, 0.1% CaCl_2_) and blocking was performed using 5% BSA in TBS (20 mM Tris HCl, pH 7.4 containing 150 mM NaCl) for 4 h at room temperature. Finally the sections were incubated with primary antibody in dilution (1 : 300) at 4°C overnight in humidified chamber. The tissue sections were washed with TBST and incubated with FITC and TRITC conjugated secondary antibody (Santa Cruz Biotechnology, USA) solution (1 : 500) for 2 h at room temperature; the nucleus was visualised by DAPI (Invitrogen). The images were observed in an Olympus microscope (IX 70, Olympus Optical Co. Ltd., Shibuya-ku, Tokyo, Japan) and a confocal microscope.

### 2.15. Lungs Wet-to-Dry Weight (W/D) Ratio

The whole lungs were removed from sacrificed mice. Each lung was blotted dry, weighed, and then placed in an oven at 80°C for 48 h to obtain the “dry” weight. The ratio of weight of the wet lung to that of the dry lung was calculated to assess tissue edema [33].

### 2.16. Myeloperoxidase Assay

The MPO enzyme activity from mouse lung homogenates was determined spectrophotometrically by measuring the absorbance at 460 nm [33].

### 2.17. Bronchoalveolar Lavage Fluid (BALF) Analysis

Bronchoalveolar lavage fluid (BALF) was collected from mice lung after administration of* E. coli*. Briefly, the left lung was intratracheally lavaged with two injections of 3 mL PBS through a tracheal cannula. BALF was centrifuged at 4°C, 1000 ×g for 10 min. The supernatant was collected for total protein analysis using the BCA protein assay kit, and the pellet was smeared onto slides for cell classification and counting with a modified Giemsa stain. Levels of TNF-*α*, IL-1*β*, IL-6, and IL-10 in the BALF were determined using ELISA kits [34].

### 2.18. Flow Cytometric Analysis

Bronchoalveolar lavage fluid cells were isolated from different groups administered with LTL and* E. coli*, stained with FITC conjugated anti-CXCL5 and CXCL8, and subjected to flow cytometric analysis using a Beckton Dickinson instrument (San Jose, CA, USA).

### 2.19. Assessment of Vascular Permeability (Lung Capillary Leakage)

Extravasation of Evans blue dye albumin (EBA; Sigma) into the tissue was used as an index of increased vascular permeability [35]. After administration of* E. coli*, Evans blue (20 mg/kg) was administered i.v. (1 mL/kg) via a tail vein 30 min prior to sacrifice. The lung tissue was incubated in formamide (4 mL/200 g lung tissue, 24 h, 37°C) and centrifuged at 5000 g for 30 min. The optical density of the supernatant was determined spectrophotometrically at 620 nm. EBA concentration was calculated against a standard curve and expressed as micrograms of EBA/gram of tissue [35].

### 2.20. Statistical Analysis

All values are expressed as mean ± SEM. Statistical significance was determined by comparing between various treatment groups and controls using the one-way analysis of variance (ANOVA). Data were considered statistically significant when *P* values were <0.05.

## 3. Results

### 3.1. TLC Analysis of LTL and Its Effect on the Production of TNF-*α* and PGE2 by* E. coli* Stimulated Mouse Peritoneal Macrophage

**Figure 1:**
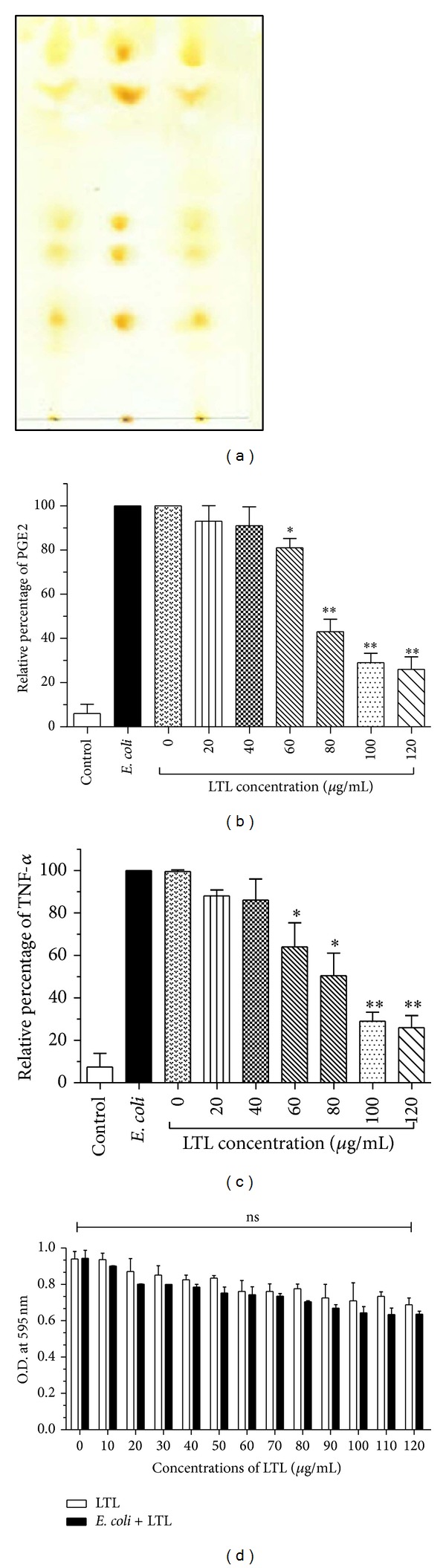
Thin-layer chromatography (TLC) profile of leishmanial total lipid or LTL (a). Effect of LTL on production of PGE2 (b) and TNF-*α* (c). The mouse peritoneal macrophage cells were preincubated with LTL in the presence or absence of heat-killed* E. coli* (O18:K1; 1 × 10^8^ CFU/mL) for 24 h and the optical density was determined by ELISA method. (d) The cytotoxicity of LTL on peritoneal macrophage cell measured by MTT assay for 24 h and OD determination at 595 nm. The data are reported as the mean ± SEM of triplicate experiments. (*P < 0.05, **P < 0.01).

Iodine staining showed six spots of lipids in the TLC plate. Lipids from three different batches showing the same TLC profile (Figure 1(a)) were used in further studies.

Macrophages contribute to the initiation of the inflammatory response in the presence of external stimuli like* E. coli*. To obtain a first insight into the anti-inflammatory role of leishmanial total lipid (LTL) isolated from* L. donovani,* LTL (0 to 120 *μ*g/mL) significantly reduced the levels of PGE2 and TNF-*α* (77.23% and 56.32% resp.) in* E. coli* treated peritoneal macrophage cells at 24 h as evident from Figures 1(b) and 1(c). Thereafter we selected the two concentrations of leishmanial total lipid, 50 *μ*g/mL (LTLd_1_) and 100 *μ*g/mL (LTLd_2_), for the* ex vivo* experiments. No cytotoxic effect was observed up to 120 *μ*g/mL of LTL as shown in Figure 1(d).

### 3.2. LTL Suppresses the Production of Several Inflammatory Mediators by* E. coli* Stimulated Peritoneal Macrophages

**Figure 2:**
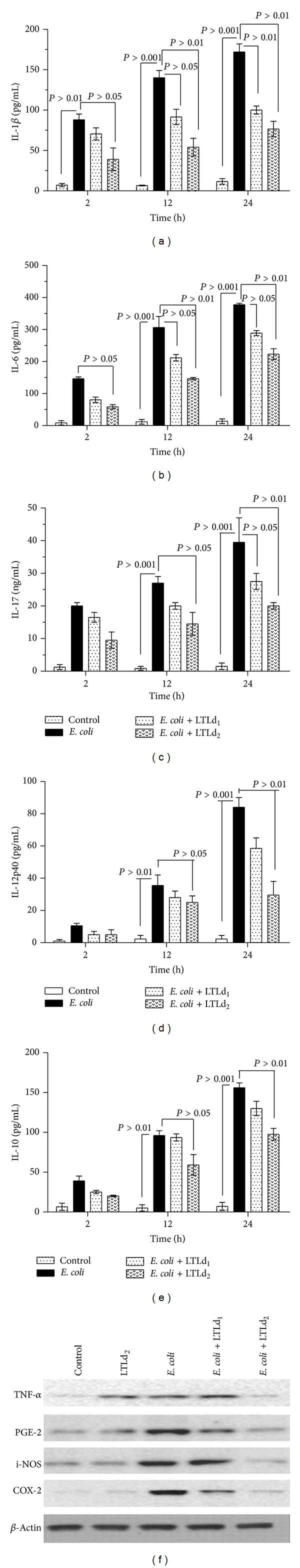
After 12 h incubation of LTL in* E. coli* induced peritoneal macrophage cells with heat-killed* E. coli* (O18:K1; 1 × 10^8^ CFU/mL) alone or with LTLd_1_ (50 *μ*g/mL) or LTLd_2_ (100 *μ*g/mL), effect on production of cytokines IL-1*β* (a), IL-6 (b), IL-17 (c), IL-12 (d), and IL-10 (e) was measured by ELISA at 2, 12, and 24 h and (f) the inflammatory mediator expression level was determined by western blot analysis. The values are mean ± SEM of three independent experiments. (*P < 0.05, **P < 0.01).

LTL has been found to subdue the inflammatory condition induced by* E. coli* in murine peritoneal macrophages. As measured by sandwich ELISA and shown in Figures 2(a)–2(f), it also significantly lowered the levels of proinflammatory cytokines including IL-1*β*, IL-6, IL-17, and IL-12 and of the anti-inflammatory cytokine IL-10 in the cell supernatant. For this experiment, the supernatant was cotreated with* E. coli* and LTL at the concentrations of 50 *μ*g/mL (LTLd_1_) and 100 *μ*g/mL (LTLd_2_) at 2, 12, and 24 h. Western blot results also revealed the impeding effect of LTL, wherein the expression levels of inflammatory mediators including TNF-*α*, PGE2, iNOS, and COX-2 were suppressed at 12 h upon pretreatment with LTLd_1_ and LTLd_2_.

### 3.3. LTL Subdues NF-*κ*B Activation and Expressions of TLR4 and CD14

**Figure 3:**
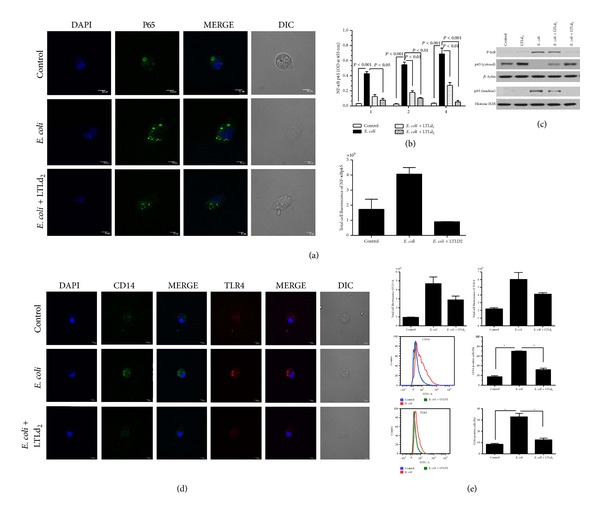
Effect of LTL on expression levels of (a) NF-*κ*B p65 in peritoneal macrophage cells after 4 h incubation at concentration LTLd_2 _(100 *μ*g/mL) in the presence and absence of heat-killed* E. coli* (O18:K1; 1 × 10^8^ CFU/mL), from immunofluorescence level measured by confocal laser scanning microscopy. (b) ELISA results showed the nuclear extract of NF-*κ*B p65 in peritoneal macrophage cells after 1, 2, and 4 h incubation with LTLd_1 _and LTLd_2_. (c) Western blot analysis revealed nuclear and cytosolic NF-*κ*B p65 levels and p-I*κ*B protein expression level after treatment with LTLd_1_ and LTLd_2_ following 12 h incubation. (d) Immunofluorescence expression measured by confocal laser scanning microscopy (magnification 600x) and (e) by flow cytometry showing levels of CD14 and TLR4 in peritoneal macrophage cells after 4 h incubation at concentration LTLd_2_ in the presence and absence of heat-killed* E. coli* (O18:K1; 1 × 10^8^ CFU/mL). The data are reported as the mean ± SEM of triplicate experiments (*P < 0.05, **P < 0.01).

*E. coli* induced inflammatory stress is known to cause activation of the transcriptional factor NF-*κ*B and the subsequent release of inflammatory mediators with signalling cascade. Thus, we examined if LTL inhibited the levels of NF-*κ*B p65 expression in a concentration and time dependent manner. As shown in Figure 3(b), LTLd_2_ significantly inhibited NF-*κ*B p65 level. Immunocytochemistry studies also provided evidence that the expression level of NF-*κ*B p65 subunit is lowered in* E. coli* stimulated macrophage cells in the presence of LTLd_2_ (Figure 3(a)). The result was validated by western blot data proving that in presence of* E. coli* stimulation, LTL suppressed the levels of both NF-*κ*B including cytosolic and nuclear portions and p-I*κ*B (Figure 3(c)). NF-*κ*B activation represents a paradigm for controlling the function of different regulatory proteins via phosphorylation based on the cascade series including proteolytic degradation of I*κ*B followed by TLR4-CD14 signalling pathway.

To explore whether LTL affects the TLR4 and CD14 molecules,* E. coli* stimulated mouse peritoneal macrophages were evaluated by an immunofluorescence study in presence or absence of LTLd_2_. The results showed that the TLR4 and CD14 protein expressions were enhanced only in* E. coli* stimulated cells; pretreatment with LTLd_2_ significantly decreased TLR4 and CD14 expression of stimulated macrophage cells at 4 h (Figure 3(d)).

### 3.4. Acute Toxicity Study with LTL

Administered intraperitoneally (i.p.), LTL was found to be nontoxic up to 500 mg/kg in mice. The experimental mice were observed for the first 24 h and monitored for the next 15 days. However, no toxic symptom or abnormal behaviour was observed. Thus, one-tenth of this dose, that is, 50 mg/kg i.p. mentioned as LTLD2, was taken as the higher experimental dose; the lower experimental dose selected was 25 mg/kg i.p. and mentioned as LTLD1. The biochemical and haematological parameters are provided in Tables 1 and 2.

### 3.5. Protective Role of LTL on* E. coli* Challenged Mice

**Figure 4:**
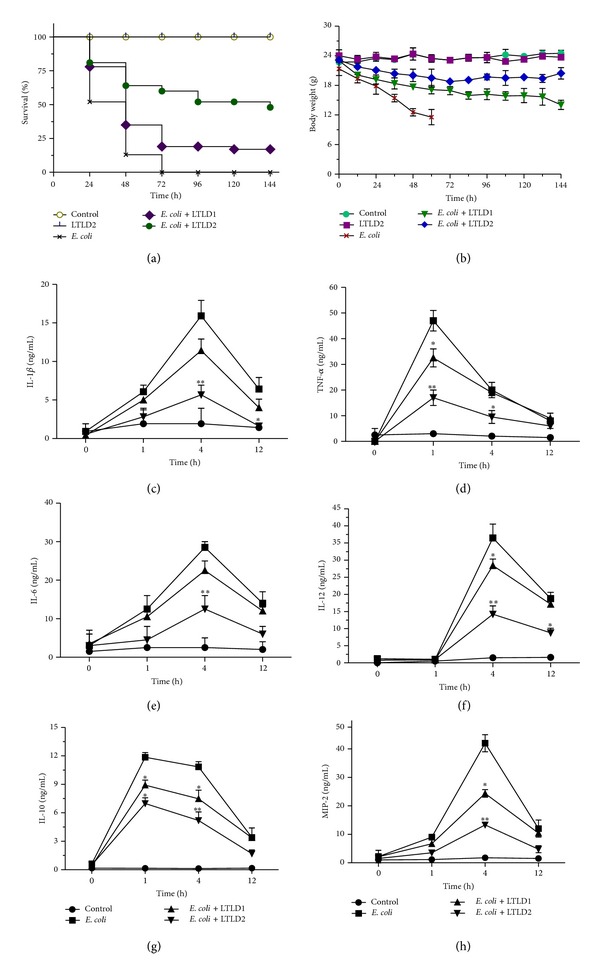
Effect of LTL on (a) survival and (b) body weight of mice (*n* = 10) treated with heat-killed* E. coli* O18:K1, 10^4^ CFU in 200 *μ*L of sterile isotonic saline. Levels of cytokines IL-1*β* (c), TNF-*α* (d), IL-6 (e), IL-12 (f), IL-10 (g), and MIP-2 (h) were measured by ELISA at 1, 4, and 12 h upon treatment with LTL at doses LTLD1 (25 mg/kg) and LTLD2 (50 mg/kg), after challenging with heat-killed* E. coli*. The data are reported as the mean ± SEM of triplicate experiments. (*P < 0.05, **P < 0.01).

The effect of LTL in* E. coli* induced death was assessed by measuring the survival rate of BALB/c mice as shown in Figures 4(a) and 4(b). In the bacterial sepsis model mortality was significantly reduced from 100% to 52.7% or 23.4% when mice were treated with two different doses, LTLD1 (25 mg/kg i.p.) and LTLD2 (50 mg/kg i.p.). Thus, the survival rate of mice improved significantly with LTLD2 compared to those receiving only* E. coli* (*P* < 0.01).

Excessive production of cytokines including TNF-*α*, IL-1*β*, IL-6, IL-12, and IL-10 and of the chemokine MIP-2, linked with a fatal outcome, was found only in serum of* E. coli* challenged mice. Conversely, pretreatment with LTL at the doses LTLD1 and LTLD2 significantly (*P* < 0.01) reduced the elevated level of proinflammatory cytokines and chemokine at 0, 1, 4, and 12 h (Figures 4(c)–4(h)).

### 3.6. Effects of LTL on Lungs in* E. coli* Challenged Mice

**Figure 5:**
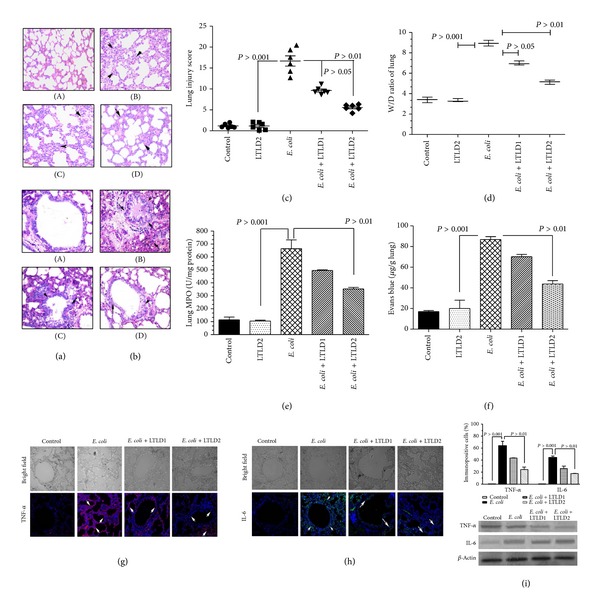
Effects of LTL on lung histopathological changes in heat-killed* E. coli* challenged mice (*n* = 10). Mice were administered i.p. with LTL at doses LTLD1 (25 mg/kg) and LTLD2 (50 mg/kg) prior to heat-killed* E. coli* O18:K1, 10^4^ CFU challenge. (A) Control group, (B)* E. coli* group, and (C) and (D) with LTL at doses LTLD1 and LTLD2. The arrows indicate infiltration generated prominent inflammatory cells and alveolar hemorrhage. Left panel (a) shows H&E staining and right panel (b) shows PAS staining, original magnification (×200). (c) The total lung injury score was calculated by adding up the individual scores of each category. (d) Lung W/D ratio assessment and the evaluation (e) of lung MPO activity with (f) the extravasation of parenchymal vascular leak. Localization of TNF-*α* (g) and IL-6 (h) in lung tissues after* E. coli* challenge in mice. Magnification (200x) arrows indicate immunopositive cells. Approximately 200 cells were counted per field, five fields were examined per slide, five slides were examined per group, and data were validated by western blot analysis (i). Values are presented as mean ± S.E.M. (*P < 0.05, **P < 0.01).

Histopathological changes like fluid and protein accumulation and the infiltration of inflammatory cells with marked swelling in alveolar wall were noted in* E. coli* challenged mice. Less infiltrate was observed in H&E and PAS stain with LTL and simultaneously alveolar wall thickening and edema were markedly reduced as evident from Figures 5(a) and 5(b); histopathological scores for mice lungs are found in Figure 5(c). As shown in Figures 5(d)–5(f), the lung W/D concentration ratio, the extravasation of parenchyma, and the lung MPO were found to be significantly lowered with simultaneous reduction of vascular permeability (*P* < 0.01 for LTLD2) at 24 h after treatment with LTL as compared to those in only* E. coli* challenge.

To evaluate the localization of proinflammatory cytokines in tissue level expression on* E. coli* induced lungs injury, TNF-*α* and IL-6 localization were checked in alveolar epithelial tissue by immunofluorescence analysis (Figures 5(g)–5(h)). Reduced expressions were observed in dose dependent manner in* E. coli* challenged mice pretreated with LTL (*P* < 0.001) as compared with only* E. coli* treated mice; the expression was estimated as the percentage of positively stained cells where it was significantly (*P* < 0.01) lowered with LTLD2 for TNF-*α* and IL-6 positive cells. Besides these, to validate our findings, we have also examined the effect of LTL at the doses of LTLD1 and LTLD2 in sepsis induced murine lung by western blot analysis (Figure 5(i)).

### 3.7. Effects of LTL on BALF in* E. coli* Challenged Mice

**Figure 6:**
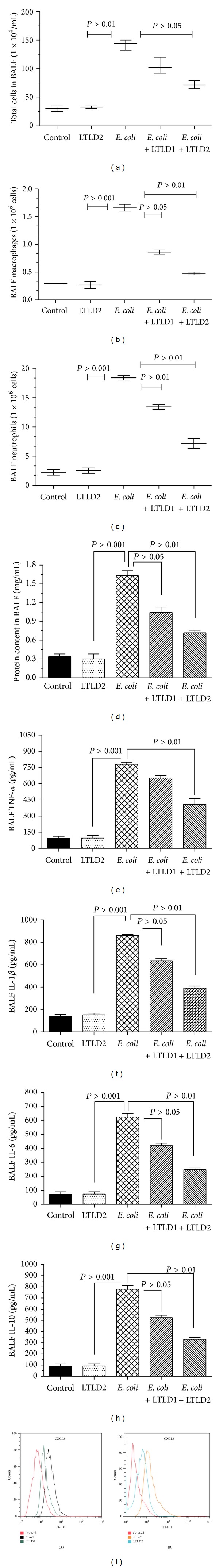
Effects of LTL on heat-killed* E. coli* induced inflammatory cell accumulation in BALF. BALF was prepared from mice 12 h after heat-killed* E. coli* O18:K1, 10^4^ CFU challenge with the induction of LTL at doses LTLD1 (25 mg/kg) and LTLD2 (50 mg/kg). Total cell counts (a), number of macrophages (b), neutrophils (c), and total protein (d) in BALF samples. LTLD1 and LTLD2 reduced the elevated level of inflammatory cytokines in BALF of* E. coli* challenged mice. BALF was collected at 12 h following* E. coli* challenge to assess the inflammatory cytokines such as TNF-*α* (e), IL-1*β* (f), IL-6 (g), and IL-10 (h) by ELISA and the chemokines CXCL5 and CXCL8 (i) by flow cytometry. Data are shown as means ± S.E.M. (*P < 0.05, **P < 0.01).

LTL at the doses of LTLD1 and LTLD2 (i.p.) was administered in mice prior to* E. coli* challenge and BALF was collected to examine different parameters. The result showed that LTL at LTLD1 and LTLD2 dosages decreased the total cell count significantly when compared with the group receiving only* E. coli* (*P* < 0.05). Preadministration of LTL caused a significant reduction in the number of neutrophils and macrophages and in total protein concentration in BALF as compared with mice exposed to* E. coli* only (Figures 6(a)–6(d)).

Simultaneously, significant differences in cytokine level were observed at LTLD2 for TNF-*α* (Figure 6(e)) and IL-10 (Figure 6(h)) (*P* < 0.01) and at LTLD1 (*P* < 0.05) and LTLD2 (*P* < 0.05) for IL-1*β* (Figure 6(f)) and IL-6 (Figure 6(g)) with* E. coli* challenge group, measured by ELISA from murine BALF at 12 h after the* E. coli* challenge. Interestingly, BALF chemokine levels of CXCL-5 and CXCL-8 were remarkably attenuated on treatment with LTLD2; significant differences are given in Figure 6(i).

### 3.8. Effects of LTL on Cell Adhesion Molecule

**Figure 7:**
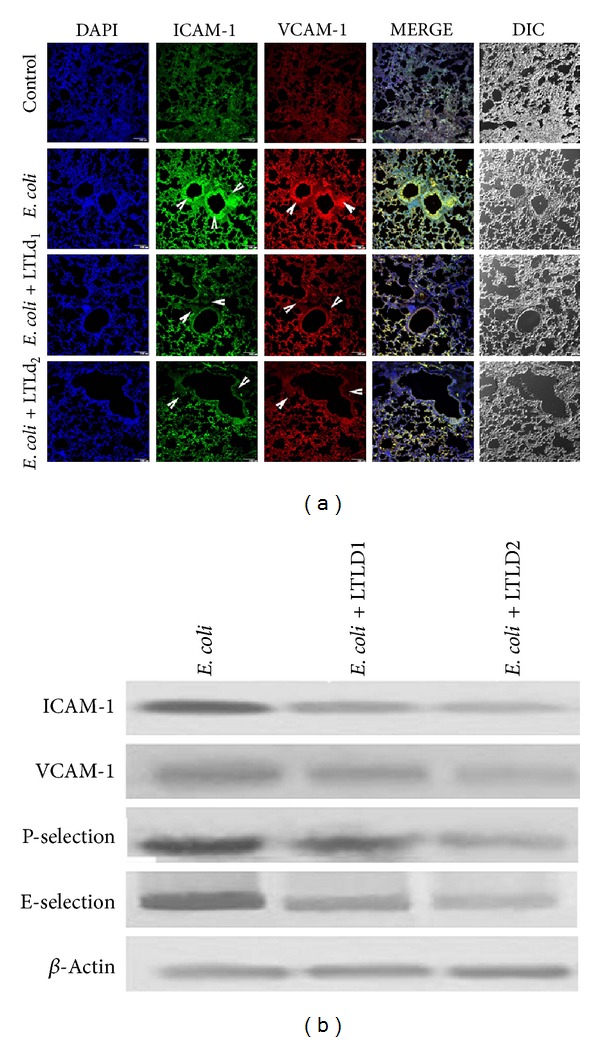
Effect of LTL in cellular localization of (a) intercellular adhesion molecule-1 (ICAM-1, green) and vascular cell adhesion molecule-1 (VACM-1, red); arrow indicates localization of cell adhesion molecule evaluated by laser scanning confocal microscope in 100x magnification. (b) Western blot protein level expression of cell adhesion; ICAM-1, VCAM-1, P-selectin, and E-selectin were markedly suppressed after* E. coli* O18:K1, 10^4^ CFU challenge and pretreatment with LTL at doses LTLD1 (25 mg/kg) and LTLD2 (50 mg/kg) in mice lung.

Inflammatory mediators and cell adhesion molecules including ICAM-1, VCAM-1, P-selectin, and E-selectin participate in inflammatory sepsis induced lung injury. ICAM-1 and VCAM-1 were used in our study to observe the effect of LTL on the lung of* E. coli* challenged mice. LTLD1 and LTLD2 were found to cause significant reduction in ICAM and VCAM-1 levels of lung epithelial tissue as compared to the only* E. coli* infected group (Figure 7(a)). Furthermore, western blot data revealed that upon pretreatment with LTLD1 and LTLD2, the protein level expressions of P-selectin and E-selectin were reduced at 24 h as seen in Figure 7(b).

## 4. Discussion

Bacterial sepsis confers the pathologic condition associated with cytokine storm, the excessive and sustained production of different cytokines by immune cells. Gram-negative bacteria, namely,* E. coli,* may trigger a life-threatening condition frequently associated with systemic dissemination of endotoxin and septic shock [36]. Host defense in bacterial infection is an established domain of the innate immune system, as a rapid response to invading pathogens is essential for survival [37]. Alteration in innate immune response directs the modulation of antimicrobial immune function with increased TLRs and CD14 responsiveness by macrophages. This heightens the susceptibility of cytokine-chemokine function and leads to neutrophil activation, initiation of tissue damage, and multiple organ failure (MOF), associated with various inflammatory diseases [38–40].

Leishmanial lipid reduces inflammatory cytokine and NO production by stimulated macrophages [41] and also induces apoptosis of synovial fluid mononuclear cells (SFMCs) through the mitochondrial-mediated pathway as reported earlier [22]. In the present study, we have demonstrated that leishmanial total lipid (LTL) exerted anti-inflammatory activities* in vitro* as well as* in vivo*.

To decipher the molecular approaches by which LTL inhibits the inflammatory responses of gram-negative bacterial sepsis, we have evaluated the survival rate and body weight improvement of mice in* E. coli* challenge murine sepsis model. Interestingly, LTL induced inhibition of production of serum cytokines including TNF-*α*, IL-1*β*, IL-6, IL-12, and IL-17 and of the chemokine MIP-2, and this was consistent with our* in vitro* results (Figure 4). This is also in agreement with our* in vitro* results (Figure 2) that this may improve the pathogenesis of bacteremic sepsis, reflected in serum profile to organ failure. TNF-*α* and IL-1*β* are known as signature cytokines that initiate an acute inflammatory cascade to cause inflammatory injury leading to the recruitment of inflammatory cells to the affected organ [42, 43]. IL-6 has been found to be the principal offender of morbidity and mortality in bacteremic sepsis [44]. In* in vitro* culture, TNF-*α* plays a central role to regulate the other cytokines especially IL-17 and IL-12 that are rapidly generated in bacterial* E. coli* infection [45, 46]. Interestingly, LTL reduced the elevated levels of these cytokines. The potent inflammatory mediator PGE2 possesses an inhibitory impact on TLR dependent TNF-*α* activation [47]. Thus, LTL attenuates the systemic inflammatory reactions and multiple organ failures [48] associated with abdominal sepsis syndrome by the involvement of inflammatory mediators. It is well known that* E. coli* provokes the signalling through its receptor cluster involving CD14 and TLR4, leading to the activation of the I*κ*B kinase complex (IKK) [49]. IKK then phosphorylates the inhibitory I*κ*B protein that is necessary for ubiquitination and degradation leading to the release and subsequent translocation of NF-*κ*B p65 into the nucleus [50]. The present study has demonstrated that LTL not only inhibited cytokine-chemokine production dose-dependently, but also activated NF-*κ*B through inhibition of I*κ*-B*α* degradation (Figure 3).

Pulmonary damage causes the disruption of epithelial integrity and leads to increased vascular permeability (Figure 5). The release of inflammatory mediators is moderated by inflamed alveolar macrophages [51, 52]. Bronchial inflammatory infiltration was evident from the presence of a large number of PAS-positive cells in the large airways and of mucus in the bronchial lumen of sepsis (Figure 5). Generally, these reactions are linked to myeloperoxidase (MPO) that is abundantly expressed in neutrophils and to the concentration of total proteins in the BALF that was reduced by LTL in the inflamed condition [53]. Thus, these results indicate that a complex network of cytokines including TNF-*α*, IL-1*β*, IL-6, and other inflammatory molecules initiates, amplifies, and perpetuates the inflammatory response. TNF-*α* and IL-1*β* stimulate the production of a variety of chemokines, namely, macrophage-inflammatory protein-2 (MIP-2), into the lungs leading to activation of neutrophils [54] in serum (Figure 4); BALF was also attenuated (Figure 6) by LTL. Leukocyte recruitment to inflammatory sites requires the coordinated expression of specific combinations of adhesion molecules. Diversity at each step of the adhesion cascade (CAMs) ensures that the appropriate neutrophils accumulate for a restricted period in response to a specific challenge [55, 56] and improve the pathophysiological condition of the host. The main endothelial CAMs involved in the inflammatory response are E-selectin and two members of the Ig-gene super family, intercellular adhesion molecule (ICAM)-1 and vascular cell adhesion molecule (VCAM)-1. The expressions of these adhesion molecules, controlled at least in part by the cytokine-inducible nuclear transcription factor kappa B (NF-*κ*B) [57], are altered by LTL as shown in Figure 7.

The present study is the first to our knowledge to demonstrate that attenuated leishmanial total lipid contributes to defense during bacteremic sepsis caused by* E. coli*, by combating the inflammatory response to infection and exaggerated inflammation related tissue injury. In the present case lung injury was reduced by the suppression of cytokine induced cell adhesion molecule and this improved the survivability with alteration of pathological changes in mice with septicemic lungs.

These studies suggest that LTL has potent anti-inflammatory activity and may represent a different approach for the modulation of inflammatory responses.

## 5. Conclusion

This study represents that leishmanial total lipid (LTL) exerts anti-inflammatory responses via regulating the inflammatory factors* in vitro* and* in vivo*. Thus, LTL reduces mortality rate of gram-negative bacteria induced septic mice. It confers protection against sepsis mediated organ damage including lung injury with alteration in levels of different cytokines, chemokines, and cellular adhesion molecules.

## Figures and Tables

**Table 1:** Effect of LTL treatment on serum biochemical parameter of mice.

Treatment	SGOT (IU/dL)	SGPT (IU/dL)	SALP (IU/dL)	Bilirubin (mg/dL)	ALT (IU/dL)	AST (IU/dL)	ALP (IU/dL)	Creatinine (mg/dL)
Control (vehicle only)	40.11 ± 9.21	36.56 ± 8.30	86.41 ± 6.33	0.86 ± 2.11	43.54 ± 8.16	92.15 ± 7.00	274.16 ± 6.16	0.93 ± 5.16
LTLD1	40.00 ± 8.01	32.98 ± 6.80	84.21 ± 2.64	0.86 ± 2.96	42.76 ± 7.36	90.61 ± 6.01	284.16 ± 7.11	0.90 ± 2.74
LTLD2	42.18 ± 4.39	35.41 ± 7.21	82.25 ± 8.86	0.90 ± 1.46	40.24 ± 6.00	90.87 ± 4.11	261.32 ± 7.21	0.91 ± 2.90

The data are reported as the mean ± SEM (*n* = 10).

**Table 2:** Effect of LTL treatment on haematological parameter in mice.

Treatment	Hemoglobin (g/dL)	RBC (10^6^/mL) cells	WBC (10^6^/mL) cells
Control (vehicle only)	14.21 ± 1.33	5.86 ± 1.05	4.21 ± 2.10
LTLD1	13.95 ± 1.10	6.36 ± 0.69	3.87 ± 2.12
LTLD2	13.92 ± 1.22	6.16 ± 1.46	3.06 ± 1.21

The data are reported as the mean ± SEM (*n* = 10).

## Abbreviations

BALF: Bronchoalveolar lavage fluid
CFU: Colony-forming unit
DAPI: 4′,6-Diamidino-2-phenylindole
*E. coli*: *Escherichia coli*
ELISA: Enzyme-linked immunosorbent assay
FITC: Fluorescein isothiocyanate
ICAM-1: Intercellular adhesion molecule 1
I-*κ*B: Inhibitor of kappa B
IL-1*β*/6/10/12p40: Interleukin 1 beta/6/10/12p40
ip: Intraperitoneally
LTL: Leishmanial total lipid
LTLd1/LTLd2: *In vitro* LTL concentration 50 *μ*g/mL and 100 *μ*g/mL
LTLD1/ LTLD2: *In vivo* LTL dose 25 mg/kg and 50 mg/kg
MPO: Myeloperoxidase
NF-*κ*B: Nuclear factor kappa B
TNF-*α*: Tumor necrosis factor-*α*
TRITC: Tetramethylrhodamine-5-(and 6)-isothiocyanate
VCAM-1: Vascular cell adhesion molecule 1.
